# Human microbiome research: Growing pains and future promises

**DOI:** 10.1371/journal.pbio.3002053

**Published:** 2023-03-17

**Authors:** Jens Puschhof, Eran Elinav

**Affiliations:** 1 Division of Microbiome & Cancer, DKFZ, Heidelberg, Germany; 2 Systems Immunology Department, Weizmann Institute of Science, Rehovot, Israel

## Abstract

The past 20 years of research has wrought huge changes in human microbiome research. This Perspective highlights the difficulties and challenges, achievements and future promises of the field.

## Teaser

Human microbiome research is evolving from describing associations to understanding the impact of bioactive strains on humans. Despite challenges, progress is being made to apply data-driven microbiome diagnostics and interventions, potentially leading to precision medicine breakthroughs in the next decade.

This article is part of the *PLOS Biology* 20th Anniversary Collection.

The past 20 years have witnessed a major shift in our understanding of the role of the microbiome in human host physiology. Pioneering studies revealed that, compared with human cells and genes, prokaryotic inhabitants contribute an equal number of cells and many times more genes to the human “holobiont,” while influencing mammalian processes ranging from digestion and metabolism to immunity, and even brain functions. In addition, changes in the microbiome have been linked to a growing number of human diseases, including cancer. These findings have led to a widespread hope that the accumulating knowledge on host–microbiome interactions would be rapidly translated into treatments for human diseases.

**Fig 1:**
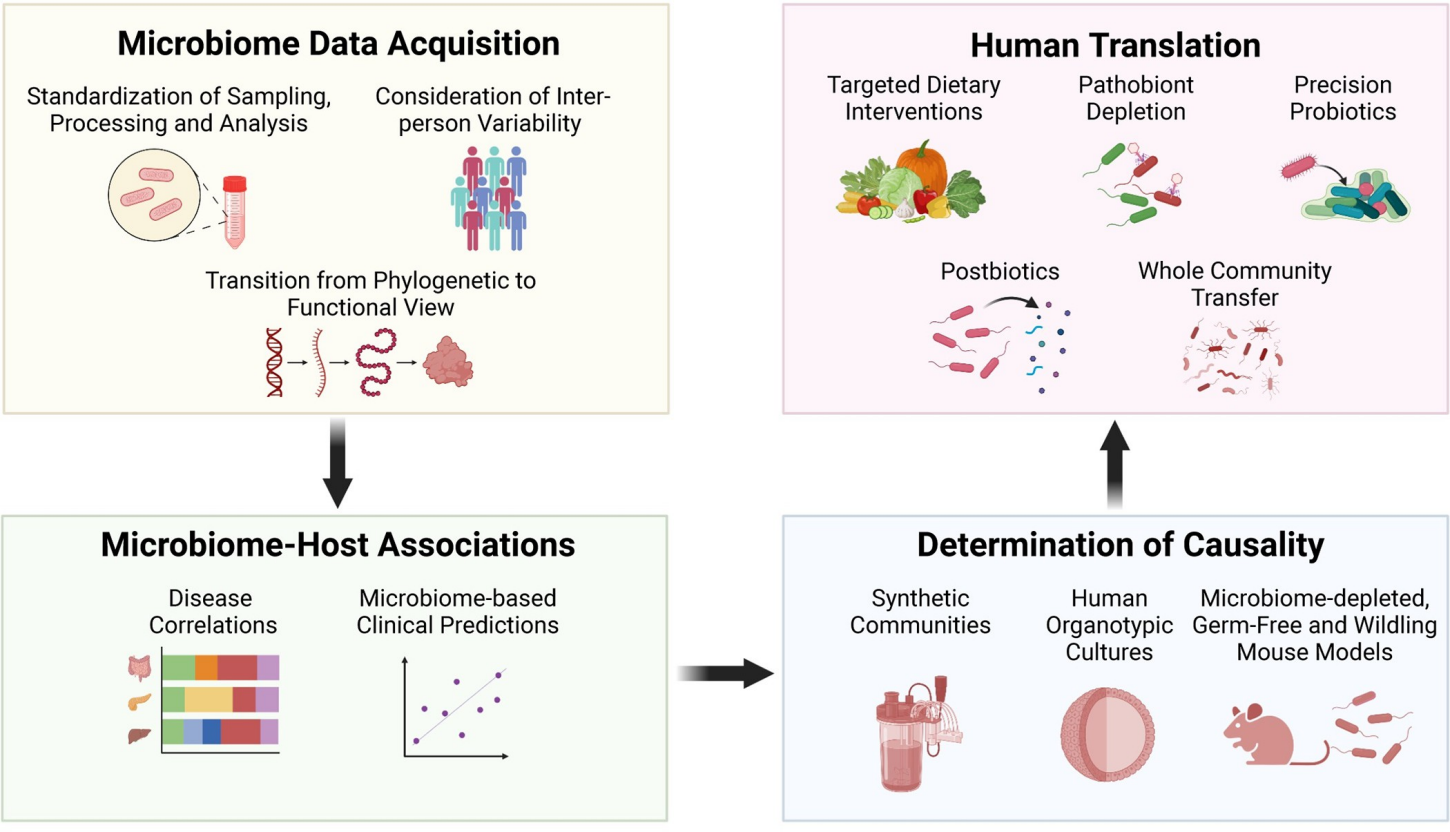
Challenges and emerging approaches in microbiome research. In meeting the formidable challenges that have complicated top-down microbiome research in the past 2 decades, the maturing field is increasingly searching for means of enhancing standardization of microbiome data acquisition and analysis, and diversifying microbiome characterization through incorporation of a variety of genomic and nongenomic pipelines, while accounting for potential confounders, caveats, and interindividual variability. The maturing field is increasingly evolving from descriptive research that highlights associations, correlations, and predictions, into mechanistic exploration of causality and molecular insights. These, coupled with enhanced usage of “big data” analytics, enable the development of personalized diagnostics and precision microbiome interventions. These modalities are expected to be tested in humans and would hopefully integrate into clinics in the coming decade. All these top-down approaches are complementary and pursued in parallel to each other and to bottom-up microbiome research.

As the field steadily matured, the associated hype that typically characterizes major scientific advances was gradually replaced with a more accurate realization of the complexity of the interactions and the numerous inherent challenges they represent on the way to rationally harnessing the microbiome in human therapy. Addressing these challenges involves a long-term, data-driven effort by many researchers. The quest is fueled by the incorporation of seemingly unrelated scientific and medical disciplines (including evolution, anthropology, and nutritional sciences, among others), development and optimization of new technologies, and identification and minimization of caveats and confounding factors, collectively aimed at achieving causal and mechanistic insights into the molecular basis of host–microbiome interactions (Fig 1). Such harmonized efforts are already yielding results that will enable more generalized and reproducible identification and modulation of exploitable therapeutic microbiome targets in the coming decade.

Indeed, the first decade of microbiome research in this millennium focused on demonstrating the importance of the gut microbiome for physiological function, coupled with next-generation sequencing-based characterization of microbiome community structure [1,2] in a variety of physiological and disease contexts. Such widespread mapping has led to the identification of associations, correlations, and predictions between the microbiome and various health outcomes, reported at ever-increasing scale and detail. In parallel, the field has extensively characterized the dynamic regulation of microbial signatures in response to a variety of factors, such as diet, host immunity [3], genomics [4], and diurnal oscillations [5]. With these substantial advances also came the recognition of major technical and conceptual obstacles challenging interpretation, generalization, and translation of microbiome findings to the clinical bedside.

One such technical challenge complicating the interpretation of microbiome-related observations is the widespread variability in sample collection, processing, and analysis methodology between different studies. Additionally, contaminations and annotation errors occurring at each step along the microbiome processing and analysis cascade may introduce artifacts and biases that are challenging to tackle and complicate the distinction between true and spurious biological signals. Understanding the scope, limitations, and confounders associated with each of these steps is essential for separating true signal from confounding factors, particularly in tissues with low or no microbial abundance [6]. Better harmonization of these methodologies, coupled with inclusion of multiple technical and biological controls, could enable more accurate interpretation of studies that can be generalized and reproduced across populations, geographies, genders, and ethnicities. However, microbiome processing and analytical standardization cannot be overly enforced without risking reductions in technological variability and diversity, which form the engines driving research innovation.

A conceptual challenge faced by most human microbiome studies is an inherent and physiologically important interindividual variability in microbiome community structures. Such person-specific microbiome signatures impose a substantial analytical challenge, as they represent an inherent and difficult-to-tackle “noise.” However, distinct microbiome configurations can be identified and harnessed to explore the microbiome contributions to individual human phenotypes, ranging from glycemic responses to supplements and foods [7] to divergent disease manifestations among individuals carrying identical disease susceptibility loci. In all, tackling the challenge of technical and biological variability using innovative methods, such as AI-based methodologies, would deepen the rational integration of microbiome knowledge into precision medicine.

An equally formidable challenge involves the progression from microbiome–human trait association to demonstration of causation. Indeed, of the many microbiota suggested to associate with, correlate with, and predict clinical outcomes, only a minority of gene products and metabolites could be shown to causally influence physiological and disease states, whereas we would expect the majority of such features to rather be altered secondary to changed environments characterizing disease states. A growing number of tools are being developed to disentangle the former, “driver” microbiome alterations, from the latter, “passenger” changes, by revealing causal and mechanistic insights. One frequently utilized approach relies on microbiota-depleted (by antibiotics treatment) or microbiota-deficient (germ-free) mouse models that allow the introduction of individual bacterial strains or communities to test their impact on host physiology. Additionally, wildling mice that harbor a microbiome similar to that of house mice living outside of captivity are rapidly gaining traction as more human-representative experimental platforms [8]. These improved in vivo models are complemented by the development of synthetic human commensal communities, improved culturing and characterization of previously “unculturable” microbes at strain-level resolution, and unbiased elucidation of bioactive microbial effectors by nontargeted metabolomics and proteomics. Organoid and organ-on-a-chip platforms are enabling the study of individual bacteria, bacterial communities, and their bioactive products in the human tissue setting [9]. With these newly acquired capacities, the field is uncovering functional readouts beyond genomic sequencing, decoding the contributions of difficult-to-culture and low-biomass microbiomes, and assessing the important, yet poorly studied, nonbacterial commensal kingdoms and their intricate interactome networks. Indeed, the expanding exploration of the potential functions of commensal and opportunistic viruses (including bacteriophages), fungi, and parasites and their impacts on the bacterial commensal ecosystem and human host constitute exciting areas of ongoing research. Investigation of these poorly characterized commensal kingdoms requires further development of research and analytical tools, including improved computational reference datasets, molecular exploitation tools, and in vivo colonization models.

Collectively, the molecular and functional insights gained by the expanding “microbiome causative toolbox” will likely enable the development of wide-ranging diagnostic, prognostic, and therapeutic human applications in the next era of microbiome research. The increasing success of fecal microbiome transplantation (FMT) approaches in and beyond the *Clostridioides difficile* infection setting is a motivating example of therapeutic microbiome use, yet failed trials and risks also underline the urgent need for increased mechanistic insights to accompany this translation [10].

Avoiding some of the field’s early technical missteps [6] while allowing innovative research to progress necessitates an appreciation of the extraordinary complexity and modularity of host–microbiome interactions, coupled with a much-needed modification of expectations. It is increasingly realized that diagnostic and therapeutic microbiome utilization in human disease will require a continued and, at times, painstaking and exhaustive exploration of molecular mechanisms and regulation, from overarching descriptive community associations to the microscale function of discrete bioactive therapeutic targets. Such exploration would likely involve an increased focus on biochemical and structural elucidation of compounds produced, modulated, and degraded by commensal microbes, their human binding counterparts, and downstream bioactive impacts on the human host.

As a positive step forward, the microbiome research community effort is already enabling the identification and exploitation of microbiome mucosal and systemic signals [11] for early detection and patient stratification by discrete disease features. Therapeutically, the naïve perception of “one size fits all” microbiome-altering dietary, probiotic [12], and community replacement interventions is gradually evolving toward a view of the microbiome as a “signaling hub.” As such, the microbiome is perceived as a context-specific relay or a buffer, integrating environmental and endogenous signals in impacting physiological and disease manifestations in individuals carrying healthy or disease-predisposing genetic traits. Such holistic realization of the microbiome as a highly modulable “fingerprint” enables the development of rational, context-specific, and data-driven interventions such as personalized nutrition, precision probiotics, metabolite supplementation (“postbiotic” therapy), and directed pathobiont suppression modalities [13]. Validation and integration of such approaches, coupled with continuous serendipitous exploration of the impact of the vast microbial communities and their associated bioactive products on the human host, will facilitate the delivery of the field’s immense promise to transform human health.
